# RNA synthesis in *Leishmania donovani* is constitutive during stage conversion: a genome-wide PRO-seq analysis

**DOI:** 10.1128/spectrum.00566-25

**Published:** 2025-06-09

**Authors:** Janne Grünebast, Stephan Lorenzen, Christine Brinker, Annika Bea, Joachim Clos

**Affiliations:** 1 Leishmania Genetics Group, Bernhard Nocht Institute for Tropical Medicine 14888, Hamburg, Germany; 2 Department of Infection Epidemiology, Bernhard Nocht Institute for Tropical Medicine 14888, Hamburg, Germany; Johns Hopkins University Bloomberg School of Public Health, Baltimore, Maryland, USA

**Keywords:** nuclear run-on, polycistronic transcription, post-transcriptional regulation, gene expression, strand switch region, amastigote

## Abstract

Transcription in *Leishmania donovani* has long been considered constitutive, as shown by nuclear run-on analyses and northern blots, with gene expression primarily regulated post-transcriptionally. Using precision nuclear run-on sequencing (PRO-seq), we investigated active transcription in axenically grown insect stages, promastigotes, and mammalian stages, amastigotes, of *L. donovani*. PRO-seq provides a high-resolution view of nascent RNA synthesis, allowing us to examine RNA synthesis rates and transcription initiation and termination sites genome-wide. Our results show uniform transcription across all chromosomes in all analyzed stages of the parasite’s life cycle. Furthermore, we observed no significant differences in transcription levels of individual polycistronic transcription units (PTUs) between promastigotes and amastigotes during differentiation after chemical or temperature/pH induction, demonstrating that environmental conditions do not influence transcription in *L. donovani*. Additionally, transcription was confirmed to be strand-specific within PTUs, with initiation and termination occurring at strand switch regions. We successfully identified sites of transcription initiation and termination, which can be used for future integration with epigenetic data. These findings reinforce that transcription in *L. donovani* is constitutive and stage-independent, excluding a role in gene expression regulation.

**IMPORTANCE:**

Our data unequivocally show that life cycle stage-dependent gene expression in *Leishmania donovani*, the parasite responsible for the lethal Kala-Azar disease, does not involve regulated RNA synthesis. We used a genome-wide analysis of RNA synthesis of cultivated parasites of three different differentiation forms to measure nascent RNA synthesis and found no significant changes. Together with earlier data, this implies mechanisms of gene expression control that set *Leishmania* apart from its human and animal hosts.

## INTRODUCTION

Leishmaniasis, classified by the World Health Organization as one of the most important neglected tropical diseases, continues to be a major global health concern, with endemic cases reported in 99 countries as of 2023 (1). Over a billion individuals, primarily in tropical and subtropical regions, are at risk of infection from protozoan parasites of the *Leishmania* genus. These parasites belong to the order *Trypanosomatida*, and they include more than 20 human-pathogenic species (1). Leishmaniasis manifests in three primary forms—cutaneous (CL), mucocutaneous (MCL), and visceral (VL)—each presenting unique clinical challenges. While CL is the most prevalent form, characterized by ulcerative skin lesions, MCL involves the destruction of mucosal tissues, and VL, the most severe form, is life-threatening if not treated. Effective management is complicated by limited drug efficacy, severe side effects, rising drug resistance, and high treatment costs (2). Despite efforts to combat the disease, including regional elimination programs, the absence of vaccines and the ongoing burden of endemic cases underline the urgent need for improved therapeutic and preventive strategies (1–3). In this study, the focus is on *Leishmania donovani*, the parasite responsible for anthroponotic VL.

The parasite’s life cycle is complex, transitioning between two distinct environments: the sandfly vector and the mammalian host (4). *Leishmania* spp. are transmitted via the bites of infected sandflies, specifically of the *Phlebotomus* and *Lutzomyia* genera (5, 6). During their life cycle, they undergo significant morphological changes from a motile, flagellated form known as promastigote in the insect vector to an ovoid, non-flagellated amastigote form inside mammalian host cells. The amastigote stage plays a critical role in the parasite’s survival. After promastigotes are transmitted into the mammalian host, they are phagocytosed by antigen-presenting cells, primarily macrophages, in which they differentiate into amastigotes. Once inside the macrophages, the parasite persists within the phagolysosome, where it replicates (7). This process not only helps the parasite to evade the immune system but also triggers inflammatory responses in the host (8). The spread of the parasite leads to the clinical symptoms of leishmaniasis, including the severe effects seen in VL. For several species, including *L. donovan*i, the differentiation of promastigotes to amastigotes can be replicated in axenic laboratory culture, providing valuable insights into the biochemical processes involved. By inducing axenic differentiation through environmental changes, such as increasing temperature from 25°C to 37°C and lowering the pH from 7.4 to 5.5, promastigotes can be transformed into amastigotes outside the host macrophage (4, 9). Furthermore, inhibiting the parasite heat shock protein 90 (HSP90) with specific inhibitors such as radicicol (RAD) or geldanamycin leads to forms similar to intracellular amastigotes (10–13).

*Leishmania* parasites and other trypanosomes regulate their gene expression post-transcriptionally, primarily by controlling mRNA stability and translation efficiency (14). Additionally, aneuploidy and smaller-scale variations in gene copy number at the genomic level influence gene expression (15, 16). This occurs because the genome is organized into polycistronic transcription units (PTUs), lacking a functional organization of genes (17–20). Early nuclear run-on studies of *Leishmania* promastigotes confirmed these PTUs on chromosomes 1 and 3. They identified the regions between two PTUs as so-called strand switch regions (SSRs), where transcription is believed to initiate and terminate (21, 22). Divergent SSRs (dSSRs), where transcription initiates, are typically characterized by acetylated histone 3 in *Leishmania major* (23), as well as by the incorporation of the histone variants H2A.Z and H2B.V in both *L. major* and *Trypanosoma brucei* (24, 25). Incorporating the histone variants H2A.Z and H2B.V instead of the core histones at dSSRs has been shown to decondense chromatin structure in *T. b*rucei and promote transcription initiation (26). Transcription termination sites, also known as convergent SSRs (cSSRs), incorporate the histone variants H3.V and H4.V in *T. brucei*; however, in *L. major*, only the histone variant H3.V has been identified (24, 25). Termination sites are further marked by Base J, a glucosylated hydroxy-methyluracil (27, 28). No transcription factors or RNA polymerase II-specific promoters have been identified in *Leishmania* spp. Therefore, transcription is thought to be constitutive, but until recently, this had only been verified for a few genes by nuclear run-on analysis in *Leishmania* promastigotes (29–32). We recently used a precision nuclear run-on sequencing (PRO-seq) approach in *L. major*, which causes CL, to look at nascent transcription on a genome-wide level, confirming constitutive transcription in the promastigote stage (33). However, we could not study the events during stage differentiation, due to the inability to induce axenic stage conversion in that species (4). A recent study in *Trypanosoma cruzi* used GRO-seq to analyze nascent transcription and found a higher transcription rate in core PTUs containing mainly conserved genes than in disruptive PTUs containing virulence genes, suggesting an influence of other factors like genome organization and cis-acting factors on transcription (34). Another recent study employed metabolic labeling of newly transcribed RNA using 4-TU (4-thiouracil) and the sequencing of purified RNA and confirmed constitutive transcription in *T. brucei* (35). They further showed that the processing of RNA and mRNA stability independently influence mRNA abundance (35). Additionally, intra-chromosomal and inter-chromosomal clustering of RNA polymerase II at transcription initiation sites in *T. brucei* seems to influence transcription initiation (36).

Here, we aimed to analyze the RNA synthesis patterns in the two distinct life cycle forms—promastigotes and amastigotes. Therefore, we used axenic promastigote and amastigote cultures and also included promastigotes treated with the HSP90 inhibitor RAD to mimic the amastigote stage. We analyzed transcription levels per chromosome and per PTU, identified transcription initiation and termination sites, and compared transcription rates between stages in *L. donovani*.

## MATERIALS AND METHODS

### Cultivation of promastigotes, axenic amastigotes, and radicicol-treated promastigotes

Promastigotes of *L. donovani* 1S were cultured at 25°C in complemented M199+ medium at pH 7.4 (Medium 199 with Earle’s salts, complemented with 20% heat-denatured fetal calf serum [FCS], 40 mM HEPES, 10 mg/L hemin, 0.1 mM adenine, 5 µM 6-biopterin, 2 mM L-glutamine, 100 U penicillin, and 100 µg/mL streptomycin). The differentiation of promastigotes to amastigotes was performed *in vitro*. On day 0, logarithmically growing *L. donovani* promastigotes were seeded at a density of 3 × 10^6^ cells/mL in 10 mL M199+ medium (pH 7.4). The cultures were incubated at 25°C without CO_2_ for 24 hours. Subsequently, the cultures were transferred to 37°C under CO_2_-free conditions. The following day, the cultures were transferred in 10 mL M199+ medium (pH 5.5) in a 25 cm² cell culture flask with a filter cap and incubated at 37°C with 5% CO_2_. On day 4, the cultures were split at a 1:5 ratio into fresh M199+ medium (pH 5.5). Complete differentiation into axenic amastigotes was achieved by day 7, and axenic amastigotes were used for PRO-seq. For radicicol treatment, logarithmically growing *L. donovani* promastigotes were seeded at a density of 4 × 10^6^ cells/mL in M199+ medium (pH 7.4). Radicicol (Carl Roth, HN72.1) was added at 1,200 ng/mL, and the cultures were incubated at 25°C for 72 hours.

### Nuclei isolation, precision nuclear run-on, and sequencing library preparation

The precision nuclear run-on and sequencing protocol follows the method outlined by Mahat et al. (37) , with some modifications implemented for *Leishmania* spp. as described previously (33). Promastigotes (log-phase), axenic amastigotes, or radicicol-treated promastigotes (three replicates each, 1.5–2 × 10^9^ cells per replicate) were pelleted (1,000 × *g*, 4°C, 15 minutes) and washed with ice-cold phosphate buffered saline (PBS). The pellet was lysed in a lysis buffer containing Tergitol 15-S-9, incubated on ice, and centrifuged. Nuclei were washed in lysis buffer without Tergitol, resuspended in storage buffer, and stored at −80°C (5 × 10^8^ nuclei per aliquot). Nuclei were mixed with an equal volume of pre-warmed 2× nuclear run-on mix, including biotin-11-CTP (1 mM) and sarkosyl, and incubated at 37°C for 10 minutes. The reaction was stopped by RNA isolation using Trizol LS. RNA was extracted using chloroform and ethanol precipitation, washed in 75% ethanol, and dissolved in DEPC-treated water. RNA was denatured at 65°C and hydrolyzed with 1 N NaOH on ice, neutralized with 1 M Tris-HCl (pH 6.8), and buffer-exchanged using P-30 columns (Bio-Rad). Streptavidin M280 beads were pre-washed and mixed with biotin-labeled RNA (using 1 mM biotin-11-CTP in the nuclear run-on) for 20 minutes at room temperature. Beads were washed with high-salt and low-salt buffers, and RNA was extracted with Trizol and chloroform, followed by ethanol precipitation. RNA was ligated with a 3′ RNA adapter after heat denaturation. A second biotin enrichment step was performed using streptavidin beads and RNA isolation, and the enriched RNA was used to prepare the downstream library. The RNA is denatured at 65°C again and incubated with a 5′ adapter in a ligation mix containing T4 RNA ligase. The reaction proceeded for 2 hours at 25°C. The RNA was purified using a third biotin enrichment, ensuring the removal of unligated adapters. The RNA was reverse-transcribed into cDNA using a specific primer complementary to the 3′ adapter. The cDNA was amplified via PCR using primers specific to the 3′ and 5′ adapters. The PCR reaction was optimized for cycle numbers to avoid over-amplification. The amplified library was purified using PAGE purification, excising libraries between ~140 bp and ~350 bp. Using the KAPA Library Quantification Kit (Roche), concentrations were measured, and sequencing was carried out on a NextSeq 550 system (Illumina) with a NextSeq 500/550 High Output Kit v2.5 (150 cycles) in single-end mode, in accordance with the manufacturer’s instructions.

### Analysis of PRO-seq data

**Fig 1:**
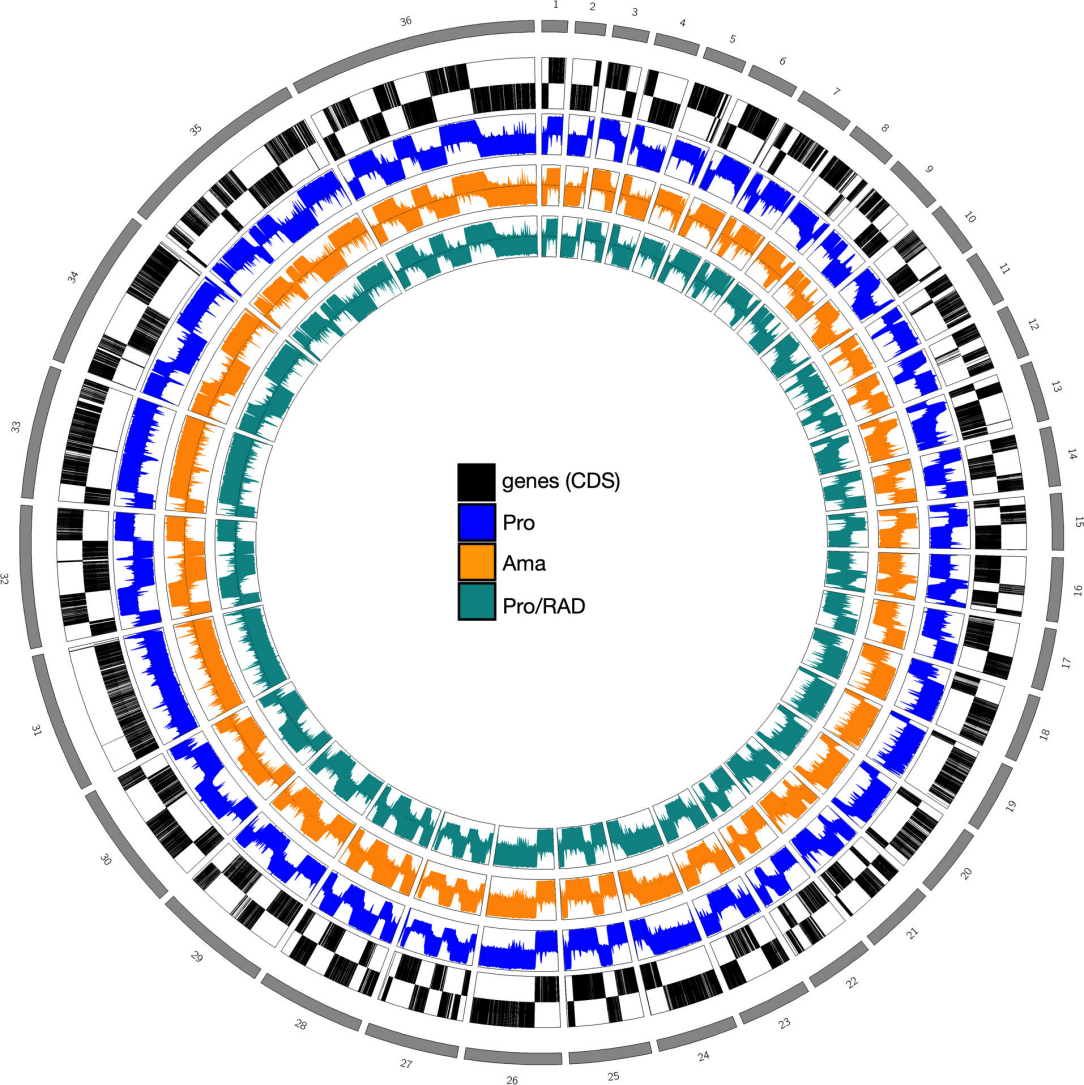
Circos plot of PRO-seq reads aligned to the reference genome *L. donovani* BPK282A1. From outside to the inside: gray represents chromosomes, black indicates genes, blue shows PRO-seq reads from promastigotes, orange reflects PRO-seq reads from amastigotes, and green corresponds to PRO-seq reads from radicicol-treated promastigotes. Genes and PRO-seq reads are plotted according to their specific strands. PRO-seq data were sequenced from three biological replicates.

The bioinformatic analysis was performed as described in reference 33. Using Cutadapt (38), PRO-seq reads were initially trimmed to remove adapter sequences and exclude short reads under 16 bp. The trimmed reads were then aligned to the reference genome of *L. donovani* BPK282A1 version 43 (TriTrypDB) using Bowtie2 (39). Bedtools (40) was subsequently used to extract the positions, and the counts were normalized according to the aligned nucleotides for each sample. PTUs were defined as sequences starting from an AUG codon and extending to the final stop codon of consecutive, unidirectional genes. The dSSRs and cSSRs were defined as regions located between head-to-head or tail-to-tail gene pairs, respectively. Base positions marked as N in the reference sequence, as well as those corresponding to RNA Pol I or RNA Pol III transcribed genes, were excluded from the calculations of mean PTU coverage. Read counts from three biological replicates were normalized and averaged. For Fig. 1 and 4 and Fig. S2 and S3, the read counts were plotted logarithmically using pseudocounts.

### Whole-genome sequencing library preparation and analysis

Genomic DNA (gDNA) from *L. donovani* 1S was isolated using the ISOLATE II Genomic DNA Kit (Bioline) and prepared for sequencing with the Nextera XT DNA Library Preparation Kit (Illumina), following the manufacturer’s instructions. In brief, fragmentation and tagmentation of 1 ng gDNA were carried out with the tagment DNA buffer and amplicon tagment mix in a thermocycler preheated to 55°C for 5 minutes. The reactions were neutralized using neutralize tagment buffer and incubated at room temperature for 5 minutes. Library amplification was performed with the Nextera PCR master mix using dual-index primers (Illumina). After amplification, the libraries were cleaned with AMPure XP beads (Beckmann) and analyzed for quality using a Bioanalyzer. Sequencing was performed on an Illumina MiSeq platform using the MiSeq Reagent Kit v3 (600 cycles). The reads were trimmed using Trimmomatic (41), aligned to the *L. donovani* genome version 1S version 45 with Bowtie (39), sorted with Samtools (42), and removed PCR duplicates. Ploidy was determined using median read density per chromosome.

## RESULTS AND DISCUSSION

A few studies have used RNA-seq analysis to examine mRNA abundance throughout the life cycle of *Leishmania* spp. and have identified genes that are differentially expressed in metacyclic promastigotes (43), amastigotes (44), and in response to heat shock (45). Differences in nascent transcription throughout the parasite’s life cycle have only been analyzed for a limited number of genes and not on a genome-wide scale; however, no increased transcription in response to heat shock was identified for these genes, leading to the conclusion that heat shock proteins are regulated post-transcriptionally (29–32). Here, we used PRO-seq (37, 46) to analyze nascent transcription in the insect and mammalian stages of *L. donovani*. PRO-seq uses an *in vitro* nuclear run-on reaction to incorporate a biotinylated NTP into the pre-mRNA during transcription (37). Subsequently, only newly transcribed mRNA is pulled down for sequencing using streptavidin beads. To examine active transcription, a method using either *in vitro* or *in vivo* incorporation of a labeled base is necessary, as traditional RNA-seq analysis typically captures mature mRNA that has undergone trans-splicing, meaning that the SL, 5′ cap, and poly-A tail have been added (14). PRO-seq allows us to truly analyze RNA synthesis rates, as well as transcription initiation and termination throughout the entire genome. In this study, we were particularly interested in examining differences in transcription between promastigotes and amastigotes, especially in view of the fact that chromatin density variations were observed in transcription start regions for amastigotes and radicicol-induced amastigotes (47). Radicicol inhibits Hsp90 in *Leishmania*, disrupting essential cellular processes and leading to the formation of amastigote-like forms (11, 48).

We used a biotinylated CTP for the nuclear run-on reaction due to the GC richness of the *L. donovani* genome. We observed successful incorporation into the pre-mRNA in promastigotes, amastigotes, and radicicol-treated promastigotes (see Fig. S1A at https://github.com/jgruenebast/Ld_PRO-seq). We performed the nuclear run-on reaction in triplicate for each stage and sequenced between 13,690,226 and 20,218,961 reads per sample (see Table S1 at https://github.com/jgruenebast/Ld_PRO-seq). For analysis, we used the reference genome of *L. donovani* BPK282A1, and between 80.60% and 89.17% of the reads mapped successfully to the reference (see Table S1 at https://github.com/jgruenebast/Ld_PRO-seq). We further checked the read length to assess potential library degradation since 20 nucleotides are protected from RNases in the RNA polymerase channel, but no degradation could be detected (see Fig. S1B at https://github.com/jgruenebast/Ld_PRO-seq).

**Fig 2:**
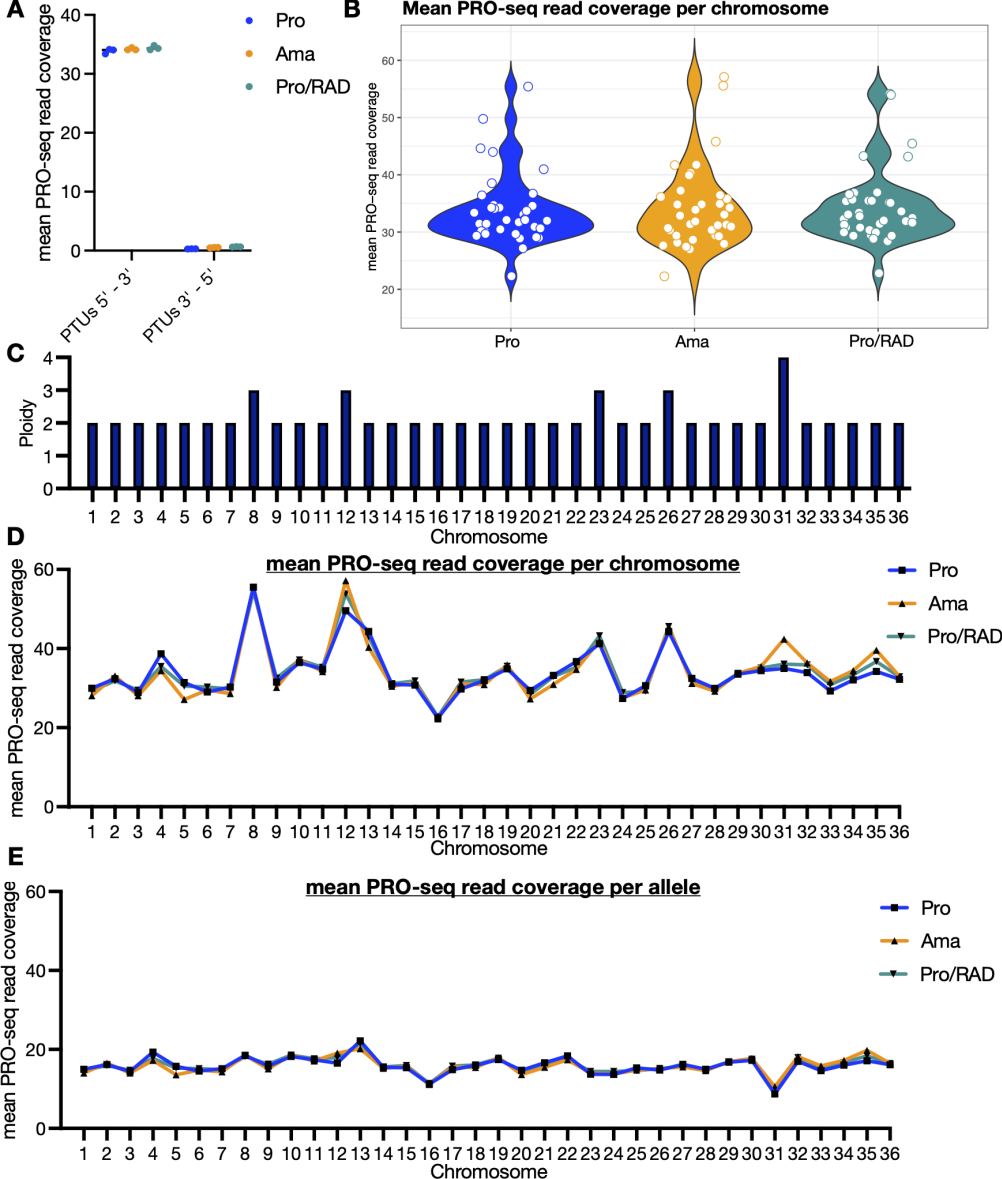
Differences in RNA synthesis levels in promastigotes, amastigotes, and radicicol-treated promastigotes across all 36 chromosomes. (A) The mean PRO-seq read coverage of PTUs for all chromosomes is plotted for the direction of transcription (5′–3′) and the opposite strand (3′–5′), *n* = 3. (B) The mean PRO-seq read coverage of PTUs across all chromosomes is plotted from 5′ to 3′, with each dot representing the coverage of a single chromosome, *n* = 3. (C) Ploidy of *L. donovani* 1S chromosomes. (D) The mean PRO-seq read coverage of each chromosome is plotted for the direction of transcription (5′–3′), *n* = 3. (E) The mean PRO-seq read coverage was corrected by aneuploidy and is plotted per allele per chromosome, *n* = 3.

We then examined the distribution of aligned reads across all 36 chromosomes of *L. donovani*, as we were particularly interested in the differences between stages, given that amastigotes have to adapt to a vastly different environment than promastigotes. The PRO-seq reads map predominantly in the direction of transcription (5′–3′), and all chromosomes are covered with reads at all stages (Fig. 1). Of the reads mapping at PTUs, we see an average coverage of ~34 reads for the direction of transcription, while on the opposite strand, the coverage is only ~0.5 (Fig. 2A; see Table S2 at https://github.com/jgruenebast/Ld_PRO-seq). This aligns with the unidirectional, polycistronic transcription shown in nuclear run-on reactions for chromosomes 1 and 3 (21, 22) and by single-strand RNA-seq analysis (28). This observation is also consistent with our previous study using PRO-seq in *L. major*, which confirmed polycistronic, unidirectional transcription on a genome-wide level for the first time (33). It also aligns with the TT-seq study, which used ultra-short metabolic labeling in combination with transient transcriptome sequencing conducted in *T. brucei*, which shows transcription to be unregulated (35). Many studies have used microarray (49) and RNA-seq (43–45) to analyze differences between promastigotes and amastigotes or under heat shock, finding variations in mRNA abundance. However, the difference between stages in the level of transcription has only been studied for individual genes using nuclear run-on analysis and northern blotting, not with modern high-throughput sequencing technologies (29–32). No difference in coverage of PRO-seq reads within PTUs averaged across all chromosomes could be detected between promastigotes, amastigotes, and radicicol-treated promastigotes (Fig. 2A).

At the chromosomal level, we noted differences in the occupancy of PRO-seq reads per chromosome (Fig. 2B). This is why we used whole-genome sequencing data for *L. donovani* 1S to address chromosomal aneuploidy (Fig. 2C). *Leishmania* parasites are known to use chromosomal aneuploidy as a gene regulation mechanism (15, 16). Typically, chromosomes in *Leishmania* spp. are diploid, but some can be triploid or tetraploid. If everything is transcribed at the same level, we should observe a higher transcription rate in chromosomes 8, 12, 23, and 26, which are triploid, as well as chromosome 31, which is tetraploid, in our PRO-seq data (Fig. 2C). Indeed, if we look at the RNA synthesis rate per chromosome, the coverage is higher at triploid and tetraploid chromosomes (Fig. 2D; see Table S6 at https://github.com/jgruenebast/Ld_PRO-seq). We therefore analyzed coverage per allele to account for chromosome aneuploidy, and we observed a similar RNA synthesis rate on all chromosomes (Fig. 2E; see Table S7 at https://github.com/jgruenebast/Ld_PRO-seq). Only chromosome 31 shows slightly lower coverage after accounting for being tetraploid, which represents the highest ploidy in this *L. donovani* strain used in the study. The mean PRO-seq coverage between chromosomes 13 and 16 and chromosomes 13 and 31 shows a minor significant difference in all stages, with *P* values <0.05 (see Table S9 at https://github.com/jgruenebast/Ld_PRO-seq). Overall, no major differences between the stages were detected at the chromosome level (Fig. 2D and E; see Table S8 at https://github.com/jgruenebast/Ld_PRO-seq).

**Fig 3:**
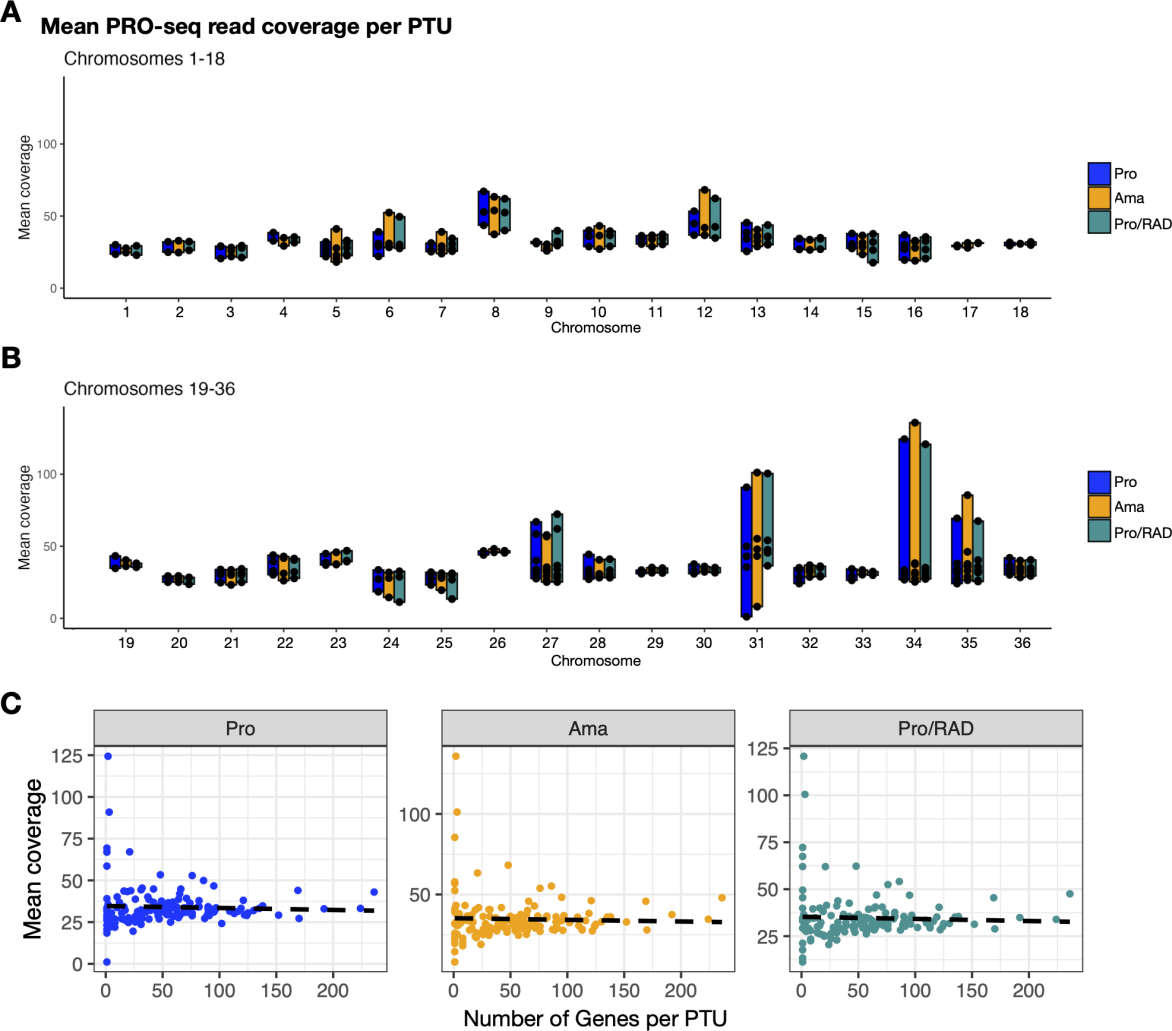
The mean PRO-seq read coverage for each individual PTU (black dots) is plotted according to the direction of transcription (5′–3′) per chromosome for *L. donovani* promastigotes (blue), amastigotes (orange), and radicicol-treated promastigotes (green), *n* = 3. (A) Chromosomes 1–18, (B) chromosomes 19–36, and (C) correlation of the mean PRO-seq read coverage of each PTU with the number of genes within that PTU, *n* = 3.

Next, we aimed to determine whether PTUs on the same chromosome have different levels of RNA synthesis across various stages. For most chromosomes, PTUs on the same chromosome are transcribed at similar levels, with only minor differences (Fig. 3A and B; see Table S3 at https://github.com/jgruenebast/Ld_PRO-seq). Chromosomes 27, 31, 34, and 35 show variations for some PTUs on the same chromosome (Fig. 3B; see Table S3 at https://github.com/jgruenebast/Ld_PRO-seq). We therefore wanted to understand if the number of genes within a PTU influences the RNA synthesis rate, but no correlation could be identified (Fig. 3C). However, the highly covered PTUs on chromosome 27 have only one gene each (1: LdBPK_072480: 60S acidic ribosomal protein P0, 2: LdBPK_072500: glycosomal phosphoenolpyruvate carboxykinase), the PTU on chromosome 31 contains only three genes (LdBPK_073400: sodium stibogluconate resistance protein, LdBPK_013350: hypothetical protein, LdBPK_013340: hypothetical protein), the PTU on chromosome 34 has two genes (LdBPK_044340: amastin-like surface protein, LdBPK_044360: D-isomer specific 2-hydroxyacid dehydrogenase), and the one on chromosome 35 includes just one gene (LdBPK_055450: pyruvate kinase). All of these highly covered PTUs are located in a telomeric region on the chromosome. The locus on chromosome 27 is adjacent to the rRNA locus, which is highly expressed. Since the PRO-seq data cannot differentiate whether reads come from RNA polymerase I or II, the high mean PRO-seq coverage at this PTU could also result from RNA polymerase I read-through. However, both genes located in this PTU on chromosome 27 play an important biological role, with phosphoenolpyruvate carboxykinase being crucial for proliferation in promastigotes and amastigotes (50), and the 60S acidic ribosomal protein P0 serving as a vital component for the assembly of the 60S subunit of the ribosome (51). Also, on the other chromosomes, the genes located within the PTUs with high mean PRO-seq coverage have an important biological function, if known. On chromosome 34, for example, the amastin-like surface protein is part of a large gene family controlling intracellular survival inside the phagolysosome, usually highly expressed in the amastigote stage (52). The PTU in which the gene is located shows similar PRO-seq read coverage in promastigotes and amastigotes, supporting the understanding that its expression is regulated post-transcriptionally. In general, both promastigotes and amastigotes show similar RNA synthesis rates for each PTU, confirming truly constitutive transcription in both the insect and mammalian stages.

**Fig 4:**
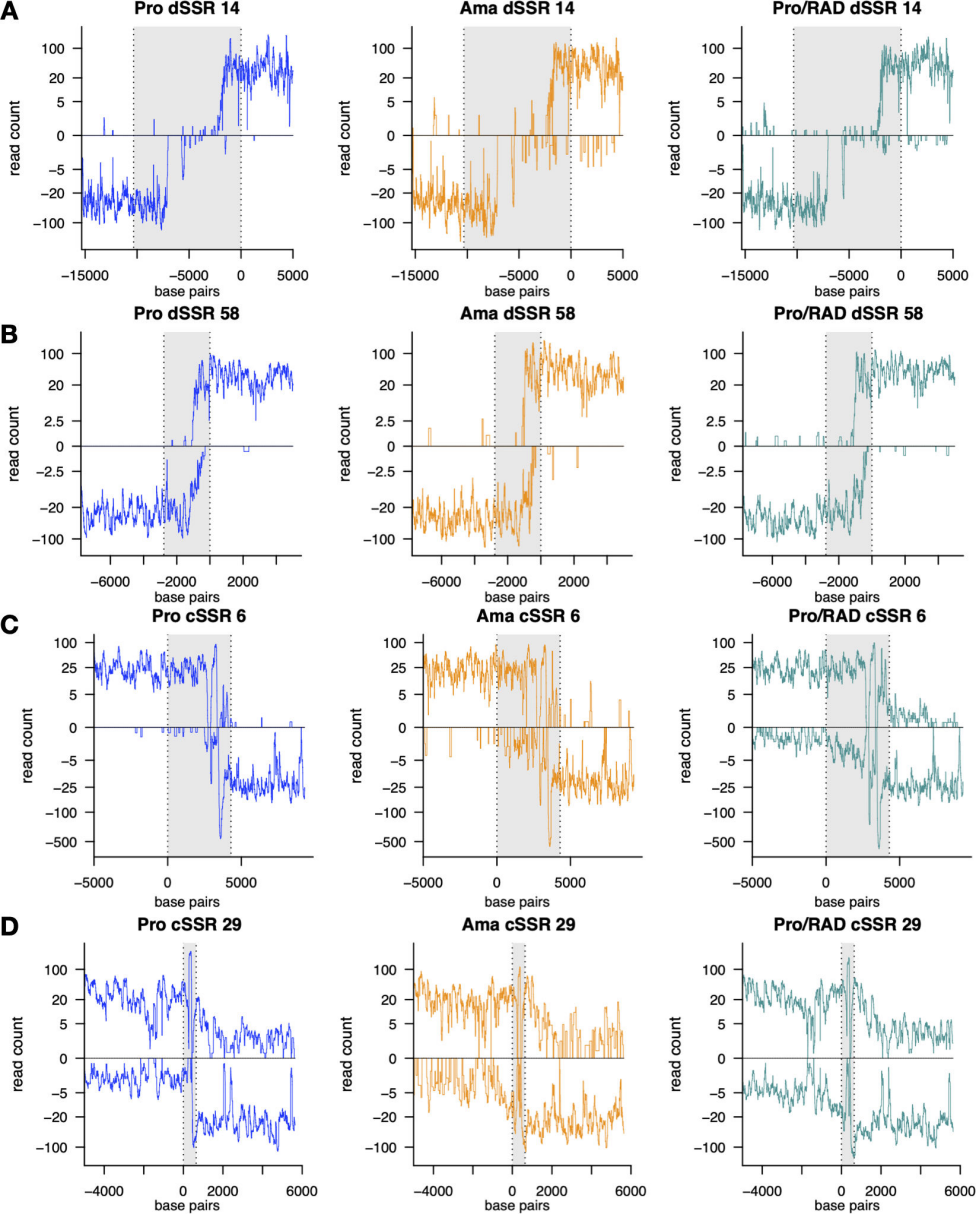
The distribution of mean PRO-seq reads of *L. donovani* at transcription initiation (**A and B**) and termination sites (**C and D**) is displayed. dSSRs and cSSRs are highlighted in gray, with dashed lines marking the start or the end of the PTUs. The first or last 5,000 bp of the PTU are plotted. Promastigotes are plotted in blue, amastigotes are plotted in orange, and radicicol-treated promastigotes are plotted in green, *n* = 3.

Both initiation and termination of RNA polymerase II-dependent transcription in *Leishmania* spp*.* show distinct characteristics compared to most eukaryotes, highlighting unique regulatory strategies in these parasites. Transcription initiation appears to occur predominantly in divergent strand switch regions, where polycistronic transcription units originate (21, 22). Those dSSRs lack canonical promoter sequences typical for higher eukaryotes, with evidence suggesting that epigenetic markers, such as specific histone modifications and open chromatin, play a central role in transcription initiation (23, 47). Among the 62 dSSRs analyzed in this study, transcription typically begins within the strand switch region for most of them (Fig. 4A and B; see Fig. S2 and Table S4 at https://github.com/jgruenebast/Ld_PRO-seq). Transcription initiation occurs in several distinct patterns: within the same region, overlapping, or with a gap in the SSR (see Fig. S2 at https://github.com/jgruenebast/Ld_PRO-seq). When comparing transcription initiation across all stages, similar patterns emerge for promastigotes, amastigotes, and radicicol-treated promastigotes, suggesting the presence of a potential underlying motif or sequence. The presence of denser chromatin in amastigotes and radicicol-treated promastigotes in some dSSRs identified via ATAC-seq does not appear to influence transcription initiation, as transcription is truly constitutive, and the patterns of transcription initiation are similar between insect and mammalian stages (47). However, the transcription initiation for most dSSRs observed in our data usually occurs over a short region, making it difficult to pinpoint the exact initiation of transcription to a base pair resolution and to search for motifs (see Fig. S2 at https://github.com/jgruenebast/Ld_PRO-seq). Research on transcription initiation in Trypanosomatids has mainly focused on *T. brucei*. One study highlighted GT-rich sequences as essential for initiation (26), while another used ChIP-seq to identify Pol II-associated conserved motifs within dSSRs, revealing distinct GT-rich and T-rich elements (53).

The process of transcription termination in eukaryotes is still not fully understood. In *Leishmania* spp., the mechanism is tightly linked to Base J, a glucosylated hydroxy-methyluracil, which plays a critical role in preventing readthrough transcription that could disrupt downstream PTUs (28, 54). Termination of transcription primarily occurs in convergent SSRs between PTUs (14). Analysis of 48 cSSRs in *L. donovani* confirmed that transcription terminates in these regions (Fig. 4C; see Fig. S3 and Table S5 at https://github.com/jgruenebast/Ld_PRO-seq), but a distinct leakage was observed for some cSSRs (Fig. 4D; see Fig. S3 at https://github.com/jgruenebast/Ld_PRO-seq). There is no existing data set for Base J in *L. donovani*, but one may speculate that Base J is absent at these sites, as previous findings in *L. major* indicate that a lack of Base J leads to a similar pattern of transcription leakage beyond cSSRs (33, 54). These patterns at transcription initiation and termination sites are comparable to the PRO-seq study we conducted in *L. major* (33).

The findings of this study highlight how *Leishmania* has developed a coordinated transcriptional strategy to efficiently regulate the transcription of large PTUs despite lacking the complex regulatory elements typical of other eukaryotes. This efficient system of constitutive transcription allows the parasite to quickly adjust the expression of essential genes in response to environmental signals using post-transcriptional mechanisms.

## Data Availability

The raw PRO-seq and whole-genome sequencing reads can be found under the SRA accession number PRJNA1220645.
